# Setting up a Governance Framework for Secondary Use of Routine Health Data in Nursing Homes: Development Study Using Qualitative Interviews

**DOI:** 10.2196/38929

**Published:** 2023-01-25

**Authors:** Yvonne Wieland-Jorna, Robert A Verheij, Anneke L Francke, Marit Tomassen, Max Houtzager, Karlijn J Joling, Mariska G Oosterveld-Vlug

**Affiliations:** 1 Nivel, Netherlands Institute for Health Services Research Utrecht Netherlands; 2 Tranzo, School of Social Sciences and Behavioural Research Tilburg University Tilburg Netherlands; 3 Department of Public and Occupational Health Location Vrije Universiteit Amsterdam Amsterdam UMC Amsterdam Netherlands; 4 Department of Medicine for Older People Location Vrije Universiteit Amsterdam Amsterdam UMC Amsterdam Netherlands; 5 Aging & Later Life Amsterdam Public Health Amsterdam Netherlands

**Keywords:** electronic health records, routinely recorded health data, learning health system, data governance, governance framework, nursing homes

## Abstract

**Background:**

In the nursing home sector, reusing routinely recorded data from electronic health records (EHRs) for knowledge development and quality improvement is still in its infancy. Trust in appropriate and responsible reuse is crucial for patients and nursing homes deciding whether to share EHR data for these purposes. A data governance framework determines who may access the data, under what conditions, and for what purposes. This can help obtain that trust. Although increasing attention is being paid to data governance in the health care sector, little guidance is available on development and implementation of a data governance framework in practice.

**Objective:**

This study aims to describe the development process of a governance framework for the “Registry Learning from Data in Nursing Homes,” a national registry for EHR data on care delivered by nursing home physicians (in Dutch: specialist ouderengeneeskunde) in Dutch nursing homes—to allow data reusage for research and quality improvement of care.

**Methods:**

Relevant stakeholders representing practices, policies, and research in the nursing home sector were identified. Semistructured interviews were conducted with 20 people from 14 stakeholder organizations. The main aim of the interviews was to explore stakeholders’ perspectives regarding the Registry’s aim, data access criteria, and governing bodies’ tasks and composition. Interview topics and analyses were guided by 8 principles regarding governance for reusing health data, as described in the literature. Interview results, together with legal advice and consensus discussions by the Registry’s consortium partners, were used to shape the rules, regulations, and governing bodies of the governance framework.

**Results:**

Stakeholders valued the involvement of nursing home residents and their representatives, nursing home physicians, nursing homes’ boards of directors, and scientists and saw this as a prerequisite for a trustworthy data governance framework. For the Registry, involvement of these groups can be achieved through a procedure in which residents can provide their consent or objection to the reuse of the data, transparency about the decisions made, and providing them a position in a governing body. In addition, a data request approval procedure based on predefined assessment criteria indicates that data reuse by third parties aligns with the aims of the Registry, benefits the nursing home sector, and protects the privacy of data subjects.

**Conclusions:**

The stakeholders’ views, expertise, and knowledge of other frameworks and relevant legislation serve to inform the application of governance principles to the contexts of both the nursing home sector and the Netherlands. Many different stakeholders were involved in the development of the Registry Learning from Data in Nursing Homes’ governance framework and will continue to be involved. Engagement of the full range of stakeholders in an early stage of governance framework development is important to generate trust in appropriate and responsible data reuse.

## Introduction

### Background: Secondary Use of Health Data

Routine electronic health record (EHR) data primarily serve to support patient care and information exchange among care professionals. In addition, routinely recorded health data are increasingly being reused for a range of secondary purposes, such as research, health policy, and quality improvement. In this manner, data from health records are reused to generate knowledge, which, in turn, can be used to improve care. By continuously reusing health records, generating knowledge, and learning from this knowledge, a learning health system can be created [1-5]. An advantage of reusing data is that it does not increase the administrative burden on health care professionals and patients.

Although researchers and policy makers widely acknowledge that reusing routinely recorded data from EHRs has great potential in creating a learning health system, they stress that a learning health system faces multiple challenges [6-9]. One of the challenges is the willingness of patients, physicians, and managers of health care organizations to share data from EHRs for secondary purposes. Trust in the appropriate and responsible reuse of data plays a pivotal role in determining whether permission is given to reuse the data. Developing and implementing a data governance framework are crucial for enhancing trust [10,11]. An adequate governance framework can be regarded as one of the several pillars of trust. In this study, we define a “data governance framework” as a set of rules and regulations determining who can use the data, for what purposes, and under which conditions. The governance framework should support the use of data in accordance with the rule of law, in an ethical, responsible, secure, and efficient way [12,13]. Although increasing attention is being paid to data governance in the health care sector, specifically in the context of a learning health system, there is little guidance on how a data governance framework should be developed and implemented in practice [8,14].

### Reuse of Data in the Nursing Home Sector

The concept of a learning health system has received the most attention in primary care and hospital settings, whereas it is still in its infancy in the nursing home sector [15,16]. However, the aging population makes it increasingly important to learn from the data recorded in nursing homes. Information about aspects such as the characteristics of nursing home residents, care provision (eg, by a nursing home physician), and quality of care for these residents could be used for scientific research, policy, and quality improvement in the sector. To address this need in the Netherlands, a registry—the “Registry Learning from Data in Nursing Homes”—is being established for the reuse of data routinely recorded in EHRs for the treatment of nursing home residents by nursing home physicians (in Dutch: specialist ouderengeneeskunde) (Textbox 1 [17]). In the Netherlands, specialized nursing home physicians are usually employed in nursing homes. They are responsible for medical treatment and care of nursing home residents. Nursing home physicians are certified after a 3-year specialty training program in medicine for older adults, in addition to their basic university training as physicians [18]. In the Netherlands, approximately 1800 nursing home physicians work for >300 different nursing home organizations [19].

**Characteristics of Dutch Registry Learning from Data in Nursing Homes [17].**

- The Registry is part of the 5-year program “Learning from Data” (2019-2024), carried out by a consortium consisting of UNO Amsterdam (University Network of Organizations for Care for Older Adults), the association of nursing home physicians Verenso, and research institute Nivel. The directors of these 3 consortium partners form the steering committee of the program.
- The data set that will be included in the Registry is a limited set of data, taken from electronic health records pertaining to Dutch nursing home residents, which nursing home physicians define as necessary for knowledge development and quality improvement.
- The data set has been defined by nursing home physicians and includes data about residents’ background characteristics, diagnosis, physical and cognitive functioning, and the care delivered by nursing home physicians.
- A trusted third party will pseudonymize the data before the data are sent and stored in the Registry Learning from Data in Nursing Homes. The database is located at and operated by research institute Nivel. The first data extraction files are expected to arrive in the first half of 2023.
- Third parties may request access to the data set for conducting scientific research. The data set will be made available for quality improvement and knowledge development in the nursing home sector by providing feedback information to nursing home physicians and enabling research.
- The governance framework and its committees are in place since July 2022.
- Since March 2022, nursing home physicians and nursing homes’ boards of directors are, in several phases, informed about the Registry and invited to participate. The Registry aims to collect data from most Dutch nursing homes.

### Legal Context

The legal context is a key determining factor in the establishment of a data governance framework. Since 2018, the European General Data Protection Regulation (GDPR) applies to the processing of sensitive data pertaining to individuals, including the reuse of personal health data for secondary purposes. Within the health care sector, patients are sometimes referred to as owners of personal and medical data pertaining to them [20,21]. Although the GDPR does not recognize data owners, patients, or in the case of the Registry, nursing home residents do have legal rights over the data pertaining to them. Furthermore, the GDPR describes the responsibilities of individuals and organizations to record and process health data (eg, nursing home physicians and nursing home organizations) [20,22]. Therefore, the Registry’s governance framework must consider the rights and responsibilities of nursing home residents, nursing home physicians, and nursing home organizations regarding circumstances and conditions in which health data may be shared with the Registry and reused for feedback information for nursing home physicians and research.

Article 9 (2) of the GDPR describes the circumstances under which secondary use of health data may be allowed. Two of these are applied to the Registry, because health data can be reused if (1) explicit consent is given by a patient or (2) processing is necessary for a scientific research aim [22]. In the Netherlands, in addition to the UAVG (Dutch GDPR Implementation Act), the reuse of health data is regulated by the Dutch Medical Treatment Contracts Act (WGBO). The WGBO applies as a *lex specialis* with regard to the GDPR and regulates whether health care providers may provide health data to researchers or others. In principle, explicit consent from the patient is required. For statistics or scientific research in public health that cannot be conducted without the data, explicit consent is not required under the WGBO in following two situations: (1) if obtaining consent is not possible, for example, if the patient is not conscious; or (2) if health care providers or others cannot be reasonably expected to ask patients for consent. In both cases, the processing should be proportionate to the aim pursued, respect the essence of the right to data protection, and provide suitable privacy protection measures such as data pseudonymization. In these specific situations, obtaining patient data with an opt-out procedure is permitted, whereby health data are reused unless a patient has objected [23]. However, demonstrating that these situations apply is not straightforward [21,24,25]. For instance, in what circumstances is asking nursing home residents for consent impossible? And in what circumstances can nursing home physicians or other nursing home staff be reasonably expected to ask for the resident’s consent?

### Guiding Principles for Data Governance

In addition to following the rule of law, other principles should be considered when developing data governance for the reuse of health data. Willison et al [26] recently published a paper on data governance conditions relevant to the reuse of data in health care settings. This was based on a literature review and consultation with key actors. The authors identified 8 guiding principles to “(1) optimize data use to meet objectives, (2) keep data secure, (3) meet privacy obligations, and (4) earn and maintain trust” [26]. Textbox 2 describes these 8 principles. To the best of our knowledge, little is known about the methodologies to adopt such governance principles while setting up a governance framework within the health care sector.

**Guiding principles for data governance as described by Willison et al [26].**

- Follow the rule of law: The governance framework should follow all appropriate legal frameworks and the governing body should ensure compliance with applicable laws, regulations, standards, and organizational policies across jurisdictions and institutions.
- Accountability: A governing body is accountable to those who will be affected by its decisions or actions. This is enforced through transparency and following the rule of law.
- Integrity: The governing process should ensure that uses of the data have a clear patient and public interest that is consistent with the intended purpose of the repository, are of high scientific and ethical integrity, and are maintained in a secure and private manner.
- Participation and inclusiveness: Patients and their families, health care professionals, and researchers should participate in governance over data use.
- Impartiality and independence: Reach a broad consensus on what is in the best interest of the patients and their families. All members of the committees must look beyond their personal interests as either patients, health care providers, or researchers.
- Transparency: All decisions, policies, and practices regarding data use are freely accessible to those affected by the decisions and to the public. These should be available in an easily understandable format.
- Effectiveness, efficiency, and responsiveness: Governance over the data repository should ensure that the objectives of the data repository are being met in an effective and efficient manner. The governing processes should serve all within a reasonable timeframe.
- Reflexivity and continuous quality improvement: Information governance should include processes that allow research to proceed in the face of uncertainty and incorporate continuous learning and quality improvement from prior experiences with data use.

### Aim

This paper aimed to describe the development of a governance framework for Registry Learning from Data in Nursing Homes, a national registry of data from EHRs on care delivered by nursing home physicians in nursing homes. The development of governance framework of the Registry is an important stage in setting up the Registry to allow the reuse of these data for research and quality improvement of care.

## Methods

### Design

The development of the governance framework for the Registry Learning from Data in Nursing Homes (Textbox 1) was guided by qualitative interviews with representatives of relevant stakeholder organizations, legal advice, consortium members’ knowledge and expertise regarding other governance frameworks, and consensus discussions among the consortium partners. The results are used to shape the rules, regulations, and governing bodies of the framework.

### Stakeholder Selection

The first step was to identify relevant stakeholders representing practice, policy, and research in the area of care for nursing home residents using the consortium members’ knowledge and expertise. As the consortium members work for organizations in research and older adults care medicine, they have a good overview of relevant stakeholder organizations in this field. Next, all potential stakeholder organizations were categorized and analyzed based on their degree of interest and power using Mendelow Matrix [27]. This resulted in the selection of 14 stakeholder organizations, who we approached with an interview request. We asked our contacts within these organizations which person could best provide us with input for the governance framework. A total of 20 participants were interviewed (Table 1).

**Table 1:** Stakeholders interviewed.

Stakeholders representing	Number and type of stakeholder organizations	Total number of persons interviewed
Nursing home residents	3 patient organizations	5
nursing home physicians and nursing professionals	1 association for nursing home physicians and 1 association for nursing professionals	4
Nursing home boards of directors	2 umbrella organizations for older adult care providers	2
Research community	4 professors associated with academic older adults’ care networks	4
Government	Ministry of Health and 1 research organization associated with the government	3
Health insurers	1 umbrella organization for health insurers	2

### Interview Procedure

Between October 2020 and January 2021, we conducted semistructured interviews with each stakeholder organization. The first and last authors (YWJ and MGOV) performed the stakeholder interviews with at least one of the other authors (RAV, ALF, MT, MH, and KJJ). Interviews were scheduled for 60 minutes. Before the interview, stakeholders received information about the “Learning from Data” program and about the scope of the interview. At the beginning of the interview, the stakeholders were asked for permission to record the interview. All stakeholders gave permission for this. Using the audio recording of the interview, a comprehensive interview report was made and sent to the stakeholders to verify whether their input was correctly reflected in the report. Subsequently, recorded interviews were deleted.

### Interview Content

The interviews started with a brief presentation regarding the background and goals of the “Learning from Data” program (Multimedia Appendix 1). Then, stakeholders were asked to share their perspectives on 3 topics (the aim and necessity of the Registry, data governance, and governing bodies). The extent to which each topic was discussed was tailored to stakeholders’ familiarity with the Registry, their knowledge of data governance, and the type of stakeholder organization they represented. We used the 8 governance principles of Willison et al [26] (Textbox 2) to guide the interviews without explicitly mentioning all principles.

1. *Aim and necessity of the registry*
  The stakeholders were informed that the Registry would be set up to make routinely recorded health data on nursing home residents available for quality improvement and knowledge development by providing feedback information to nursing home physicians and enabling scientific research. Stakeholders were invited to reflect on the aims and necessity of such registry.
2. *Data governance*
  The concept of data governance and the applicable legal context were introduced. Stakeholders were informed that researchers, policy makers, and other interested parties would be able to request access to the data set for research purposes, and that a governance framework would be put in place describing policies and procedures to determine who may have access to the data, under what conditions, and for what purpose. Documentation describing the framework would be published on the internet. Furthermore, the Registry would adopt privacy measures. These measures include data pseudonymization, a secure repository, and a procedure in which nursing home residents and their representatives can decide whether information from the EHR pertaining to them can be included in the Registry. Stakeholders were asked to share their views on who could access the data, under what conditions, and for what purpose.
3. *Governing bodies*
  Governance procedures will be carried out by governing bodies that have accountability or control over some aspects of governance. Stakeholders were asked to share their perspectives on which organizations should be invited for a position in a governing body and how they envisaged their role in such bodies.

### Interview Analysis

Interview reports served as the basis of the analysis. The interviews were conducted within the cyclic process of data collection, analysis, and new data collection. This cyclic process made it possible to refine the interview guide and further explore important themes in subsequent interviews. The reports were deductively coded by the first and last authors (YWJ and MGOV, respectively). Discrepancies in coding by the authors were resolved through discussions between YWJ and MGOV. The coding categories were based on the 8 governance principles shown in Textbox 2.

### Development of the Governance Framework

During several meetings, members of the 3 consortium partners discussed the results of the interview analysis and the draft of the governance framework to reach a consensus about the implications of the governance framework for the Registry Learning from Data in Nursing Homes. In addition, we consulted lawyers from 2 lawyer’s offices for a review of the legal basis of the Registry. In an iterative process, the results were fleshed out by the rules, regulations, and governing bodies of the governance framework.

### Ethical Considerations

According to Dutch legislation, ethics approval for this study was not required, because it did not concern medical scientific research, and did not make participants subject to procedures or required them to follow rules of behavior [28].

## Results

### Overview

In the following sections, we first describe stakeholders’ perspectives on the aim of the Registry Learning from Data in Nursing Homes. Next, we report the aspects of the data governance framework for the Registry that stakeholders found important regarding who may access the data, under what conditions, and for what purpose. Next, we discuss stakeholders’ views on the parties involved in a governing body. Finally, we describe how we used the findings to develop a governance framework for the Registry.

### Perspectives on the Aim of the Registry

All stakeholders, who were interviewed, agreed on the necessity of a national registry that includes data from the EHRs of nursing home residents because of the potential benefits it has for quality improvement and knowledge development. Stakeholders agreed that, currently, relevant information about nursing home care, such as average duration of stay, cannot easily be obtained at national or regional levels. Stakeholders supported the reuse of routinely recorded health data as feedback information for nursing home physicians and for research. Nevertheless, interviewees indicated that the scope and number of variables in the data set are determining factors in whether these aims could be achieved. The data set included in the Registry Learning from Data in Nursing Homes is a limited set of data that nursing home physicians consider as necessary for knowledge development and quality improvement. In particular, stakeholders with a scientific background pointed out that such a data set might be too limited for scientific research purposes. They were worried that a very limited data set would adversely affect the extent to which valid and representative outcomes could be generated, which is important to enhance the principle of integrity. Regarding the focus on nursing home physicians, some stakeholders mentioned that nursing home residents always receive multidisciplinary care. They argued that from a patient-centered perspective, data on care provided by other professionals working in nursing homes are needed to fully realize quality improvement for residents. We explained that the Registry will continuously evolve as it is part of a learning health system. The stakeholders welcomed this idea.

### Perspectives on Data Governance

Stakeholders acknowledged that data governance is important and predominantly shared their perspectives on how nursing home residents could exercise their rights regarding data and procedures to guide data usage.

#### Control Over the Data

In almost all interviews, Willison’s principle to “follow the rule of law” was an important topic. Stakeholders emphasized that nursing home residents, nursing home physicians, and nursing home organizations should be able to exert control over the data that pertain to them. Therefore, they mentioned that it is important that these groups are represented in the governance framework, for example, by involving authorized representatives in the governing bodies. This is also in line with Willison’s principle of “participation and inclusiveness.”

Stakeholders from all types of organizations mentioned that nursing home residents should have control over data containing personal and medical information by expressly consenting (opt in) or objecting (opt out) to the inclusion of the data pertaining to them in the Registry. Opt-in or opt-out procedures are required not only to comply with the GDPR and WGBO, but also to build trust in the appropriate reuse of data in the Registry. However, stakeholders representing patients in particular expressed concerns about such procedures. First, they were concerned by the fact that patients currently receive an increasing number of opt-in or opt-out requests for data sharing, which may impose a substantial administrative burden on patients and their health care providers. Second, stakeholders explained that many patients have difficulties understanding the information provided by the request to share data or to participate in the research. For nursing home residents with cognitive limitations, this is even more challenging. For these residents, representatives are often those who should be asked for consent. Stakeholders emphasized the need to present understandable information to nursing home residents and their representatives about which data will be used, how it will be used, and for what purposes. Thus, transparency can be enhanced. However, in the case of the Registry, such detailed information is not available beforehand, as the data can be used for multiple research questions within the aims of the Registry. Therefore, stakeholders advised carefully considering what information can be provided on the purposes and conditions of data reuse, such as the aims of the Registry.

#### Data Use and Assessment Criteria

Stakeholders pointed out that the data in the Registry should be used exclusively for purposes in conformity with the aim of the Registry to develop knowledge and to improve the quality of care provided by nursing home physicians. Unambiguous criteria are required to assess whether a request for data is in line with these aims. Stakeholders advised including the specific characteristics of the requesting party as part of these criteria. Stakeholders were particularly concerned about requests from for-profit companies, national health care system governance bodies, and organizations to which the Dutch Act on Public Access to Government Information (Wob) applies. For example, stakeholders expressed concerns about whether research commissioned by a pharmaceutical company is in the best interests of nursing home residents and their staff. In addition, stakeholders pointed out that the Dutch Health and Youth Care Inspectorate (IGJ) should not have access to the data because the supervisory role of the IGJ is not compatible with the aim of the Registry. Furthermore, as the IGJ is authorized to impose measures on facilities providing low-quality health care, providing access to the data to the IGJ might reduce trust of nursing home physicians and nursing home boards of directors in the Registry. Finally, stakeholders were worried that organizations might be forced to publish data under Wob, posing a personal privacy risk for residents and a risk of breach in confidentiality of nursing home physicians and nursing home organizations. Hence, providing access to the data to these types of organizations raises questions and concerns regarding trust and a safe learning environment. Incorporating adequate, publicized assessment criteria will contribute to the principles of “integrity” and “transparency.”

In addition to assessment criteria regarding the requesting party and the purpose of data reuse, stakeholders stated that the process of reviewing data requests should include a methodological assessment. The methodological assessment should include an examination of whether the data set is suitable for answering the proposed research question and assessing the privacy measures. Using assessment criteria for methodological aspects can stimulate integrity and transparency. In some interviews, stakeholders suggested setting up a committee primarily engaged in the assessment of privacy risks.

Periodic evaluation of governance procedures and policies as well as the evaluation and further development of the data set, were frequently mentioned. These can be regarded as aspects of “Reflexivity and continuous quality improvement.” Stakeholders noted that regular meetings of the members of the governing bodies are needed to further develop the rules and regulations of the Registry and to gain and maintain trust among members of the governing bodies.

“Effectiveness, efficiency, and responsiveness” issues were mentioned least often. Only the Ministry of Health, Welfare, and Sports expressed a need to respond quickly to public health issues within the nursing home sector.

### Perspectives Regarding Parties to Be Represented in Governing Bodies

#### Overview

All stakeholders supported the idea that governance procedures should be carried out by one or more governing bodies that are accountable and responsible for ensuring that the data are used in the best interests of nursing homes, their residents, and nursing home physicians. The question of which organizations to be involved in governing bodies was discussed in all the interviews. In 11 interviews, stakeholders commented on whether they personally or a colleague of their organization would want to serve as members and explained their perspective on which other organizations should be members. Three other stakeholders only provided their views on their own positions. Stakeholders mentioned that careful consideration is required regarding which parties could best represent groups with rights and responsibilities with respect to the data and groups who may want to use the data. Multiple stakeholders could qualify for a governing position, but only a limited number of members could be included in the bodies. In the following sections, we describe the stakeholders’ perspectives on these points.

#### Nursing Home Physicians and Nursing Home Boards of Directors

Stakeholders advised inviting a representative of nursing home physicians to take a governing position to safeguard the inclusion of the physicians’ perspective on secondary use of the data. Additionally, one or more representatives of the nursing home board of directors should be included. All the data in the Registry were recorded within the context of a nursing home, making the board of directors responsible for the data under the GDPR. Both nursing home physicians and boards of directors of nursing homes should benefit from the data through feedback information and scientific publications. If they have a position within a governing body, they will be able to provide input on which information they would need.

#### Involvement of Nursing Home Residents

Stakeholders placed a high value on the involvement of nursing home residents in the governance of the Registry, because the EHRs contain information about the nursing home residents and their treatment. Eight stakeholders proposed inviting organizations representing patients for a position in a governing body, whereas a few others proposed inviting nursing home residents. Stakeholders had different perspectives on which tasks and responsibilities are most suitable for organizations representing residents within the governance framework. The answers ranged from an advisory role, for instance, providing advice on the focus and goals of the Registry, to direct involvement in all data request assessments.

#### Scientific Representation

Stakeholders indicated that governing bodies should include members with scientific backgrounds. These members could provide input regarding methodological aspects and assess whether a request for data is in line with the developments and scientific information gaps in the nursing home sector and medicine for older adults. Stakeholders suggested inviting one or more professors of older adult care or representatives of the University Network of Organizations for Care for Older Adults. However, because each professor might have their own perspective on topics, some stakeholders wondered whether a small selection of professors could be a representative delegation. Furthermore, because the academic networks only represent a subset of Dutch nursing homes, stakeholders questioned whether the interests of nursing homes would be fully represented.

#### Representation of Other Parties Within an Expert Committee

As care for nursing home residents is provided by a multidisciplinary team, half of the stakeholders suggested involving other disciplines within a governing body, in addition to nursing home physicians. Most of these stakeholders recommended inviting the branch organization for nursing staff to play an advisory role. Other relevant parties mentioned by some of the interviewed stakeholders were the Dutch National Institute for Public Health and the Environment, the umbrella organization of health insurers in the Netherlands, and the Ministry of Health. They could use their position to keep the Registry informed about related issues in the nursing home sector, for instance, regarding policies to promote scientific research or quality improvement.

### Governance Framework for the Registry Learning From Data in Nursing Homes

#### Overview

The interviews yielded valuable information that could be used to develop a governance framework. However, incorporating this information into a governance framework required additional effort, because not all stakeholders’ views were consistent, for example, regarding whether or not academic networks should have a role in assessing the scientific quality of data requests and regarding tasks and responsibilities of representatives of nursing home residents within the governance framework.

Moreover, some advice provided by stakeholders required more elaboration before it could be readily used in the Registry’s governance framework, for example, regarding a data approval procedure. Additional efforts were required to position the different governing bodies with regard to each other. The development of the governance framework was, therefore, an iterative process and the outcome of several consensus discussions among consortium members in which the results of the interviews, as well as with legal advice and consortium members’ knowledge and expertise regarding other governance frameworks and relevant stakeholders were taken together.

**Figure 1:**
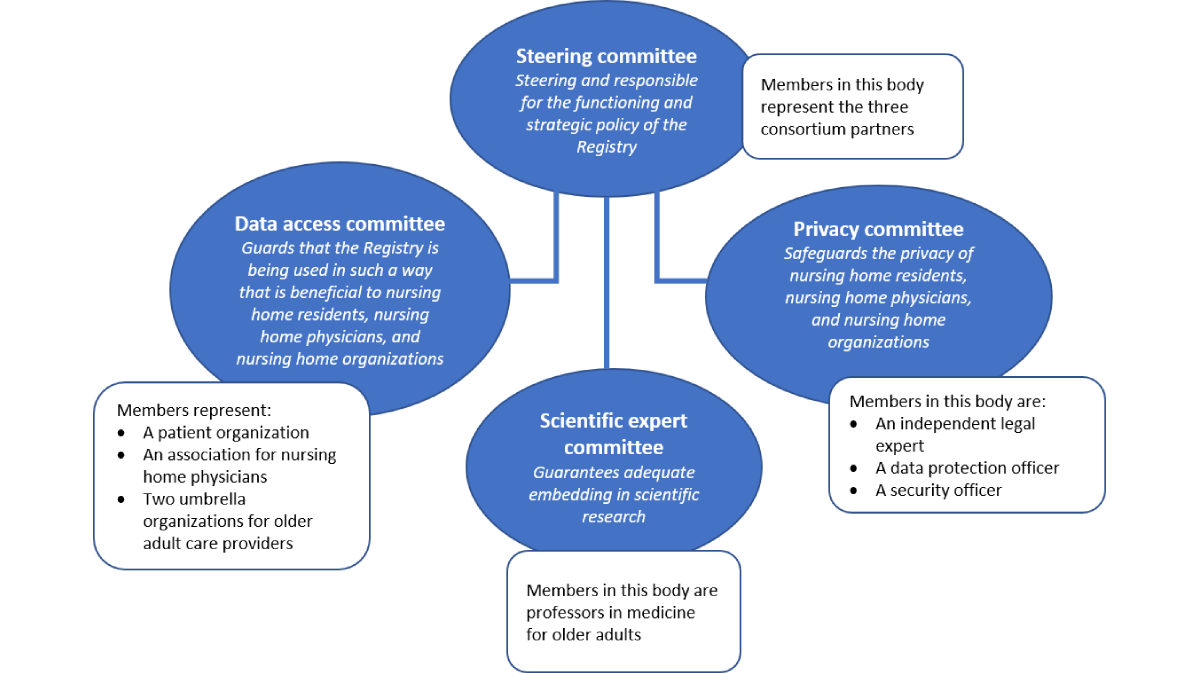
Governing bodies for the Registry Learning from Data in Nursing Homes.

The final governance framework included rules and regulations implemented by 4 governing bodies consisting of a steering committee, data access committee, privacy committee, and scientific expert committee (Figure 1). Four separate committees allow the members of each committee to focus on a particular theme (eg, privacy) and operate independently. We describe the organizations that were invited for one of the 4 governing bodies. The organization determined along with consortium members, which individuals from the organization could be invited to particular governing bodies. The organization names were published on the Registry's website [29]. Cross-representation across committees was limited. The Nivel project manager will attend meetings of all 4 committees and function as a link between the committees. For each committee, we briefly described how the tasks and composition were shaped by the iterative process mentioned above. Table 2 shows how each of the 8 guiding governance principles are fleshed out in the rules and regulations of the governance framework.

**Table 2:** Incorporation of governance principles in the governance framework of the Registry.

Principle	Rules and regulations of the Registry Learning from Data in Nursing Homes^a^
Follow the rule of law	To enhance compliance with the laws (General Data Protection Regulation and Dutch Medical Treatment Contracts Act), the privacy committee will be responsible for: Advising the steering committee about: Data requests that might pose a privacy risk for nursing home residents, nursing home physicians or nursing home organizations, for example if the data set is to be linked to an external data set; Consequences when laws or regulations change; Whether intended changes in the governance document, privacy statement, contracts, or data set are in line with legislation. Conducting regular audits to check the governance processes. In addition, the following measures are taken to enhance compliance with the relevant laws: Data from electronic health records are pseudonymized before being sent and stored in the secure repository of the Registry. A privacy statement describes the technical and organizational privacy protection measures. This includes a procedure whereby nursing home residents can exercise their rights. The boards of directors of the nursing homes and the nursing home physicians will receive information on the applicable legal aspects and will be asked whether they support the implementation of an opt-in or opt-out procedure.
Accountability	Ultimate accountability of the Registry lies with the steering committee: The steering committee is the highest governing body. The steering committee is responsible for the final decision on approval of data requests based on the assessment of the data access committee and privacy committee.
Integrity	The integrity of the reuse of data is addressed by: The data access committee, which is responsible for a data request approval procedure based on predefined assessment criteria. The procedure is designed to stimulate that: Data reuse by third parties is in line with the aims of the Registry and is being used in such a way that is beneficial to nursing home residents, nursing home physicians, and nursing home organizations; The privacy of data subjects is protected. The scientific expert committee, which will maintain close links with research communities. This will help achieve the Registry’s aim to contribute to public and scientific issues regarding care provided by nursing home physicians in nursing homes. Scientific experts within this committee advise the steering committee about developments in older adult care and related research.
Participation and inclusiveness	Participation and inclusiveness are served by: The involvement of parties in governing bodies representing nursing home residents, nursing home physicians, boards of directors of nursing homes, and professors in medicine for older adults. The fact that the steering committee will inform and consult the other governing bodies on a regular basis.
Impartiality and independence	All committee members are required to act impartially, putting aside their personal or professional interests, to reach consensus in the best interests of nursing homes, their residents, and physicians. If members of a committee come to different conclusions regarding the approval of a request for data from a third party, the chairman tries to come to an agreement. Members of the data access committee and privacy committee are not allowed to be involved in the assessment of their own requests for access to data.
Transparency	Transparency is incorporated in multiple rules and regulations. They include the following measures: The governance framework, privacy statement, assessment procedure, and content of a contract between a nursing home and the data processor (ie, Nivel) are published on the internet. All granted requests for data are published on the website of Nivel. Nursing home residents and their representatives receive information on the Registry that is complete and in an understandable format. The boards of directors of nursing homes and nursing home physicians receive information on the aim and scope of the data set in the Registry, governance framework, privacy statements, and contracts. In addition, they are offered the opportunity for an individual meeting to ask any questions they might have. Research conducted with data from the Registry will always be published in an open access publication.
Effectiveness, efficiency, and responsiveness	To enhance that the aims of the Registry are being met in an effective and efficient fashion way: Each year, the steering committee determines a plan of action in consultation with the other 3 governing bodies.
Reflexivity and continuous quality improvement	Continuous learning from developments in the nursing home sector, prior experiences with the governance framework, and the Registry’s data reuse is stimulated by: Periodic evaluation of the governance rules and regulations, scope and content of the data set, and reuse of the data for research and feedback information for nursing home physicians; The fact that all governing bodies provide advice to the steering committee on relevant themes, for example developments in the nursing home sector, research, privacy, and ICT, either at the request of the steering committee or on their own initiative.

^a^Most rules and regulations relate to more than one principle. Activities are shown according to the most dominant principle.

#### Steering Committee

The steering committee is the highest governing body and accountable to those who will be affected by the decisions or actions regarding the Registry’s functioning and strategic policy. The steering committee can be held accountable for the Registry’s functioning and strategic policy by the Dutch Data Protection Authority.

Discussions among consortium partners centered around the scope and tasks of this committee, as stakeholders did not provide explicit and unambiguous input on how assessments of data requests should be positioned in the framework. In an early draft of the governance framework, the consortium partners assigned responsibilities for strategic decisions as well as for the data approval procedure to a central committee, consisting of consortium members, 2 organizations representing the board of directors of nursing homes, and an organization representing patients in general, including nursing home residents. Later, the consortium members decided to set up 2 separate governing bodies, namely, a steering committee and a data access committee (Figure 1), with a primary reason that, due to the programs’ design (see Textbox 1), only consortium partners can be held legally accountable for the Registry. Therefore, the responsibility for the development and maintenance of the Registry was assigned to a steering committee consisting of members representing consortium partners. The involvement of consortium partners in the data approval procedure is shaped by their ultimate accountability; the steering committee provides the final decision on the approval of data requests based on the assessment of a data access committee and privacy committee.

#### Data Access Committee

The data access committee (Figure 1) is set up to ensure that the Registry is being used in such a way that is beneficial to nursing home residents, nursing home physicians, and nursing home organizations, by assessing requests from third parties that want to reuse data for scientific research and advise the steering committee on strategic decisions.

Interviewees placed a high value on the involvement of representatives of nursing home residents in any governing body, but concretization of their tasks and responsibilities could not be fully discerned from the interview results. Following the advice of most stakeholders, consortium partners decided to invite an organization representing a broad range of patients to participate in the data access committee, as this organization has experience in comparable procedures in other governance frameworks. Furthermore, an association of nursing home physicians and 2 umbrella organizations representing nursing home boards of directors were invited. To incorporate the methodological assessment suggested by stakeholders, all members of this committee require to be familiar with conducting scientific research.

#### Privacy Committee

Safeguarding the privacy of nursing home organizations, their residents, and nursing home physicians is an important point of attention raised by the stakeholders who were interviewed. A few stakeholders made this concrete by advising the Registry to set up a committee primarily engaged in the assessment of privacy risks. Consortium partners agreed that setting up such a committee for the Registry’s governance framework contributes to minimizing the risk of privacy violations and consequently helps to gain and maintain trust. In addition, this committee could enhance compliance with applicable laws. Therefore, an independent legal expert, a data protection officer, and a security officer with experience in the reuse of health data were deemed suitable for a position in the privacy committee to cover a broad range of expertise on privacy safeguards (Figure 1). The consortium members discussed whether an organization representing patients should also be invited to the privacy committee. However, as participation in both the data access committee and privacy committee was supposed to be too much, it was seen as more important that nursing home residents would be represented in the data access committee and that patient organizations could nonetheless be asked for advice on privacy matters when needed.

#### Scientific Expert Committee

Discussions regarding this committee focused on its role and the number of organizations that should take place in it. Early drafts of the governance included a large “advisory committee,” including a broad range of stakeholders whose task should be to advise the steering committee about (1) relevant issues in the nursing home sector and (2) needs and developments in scientific research in older adult care medicine. However, during the iterative process, consortium members concluded that input on the first topic could also be obtained from existing relations between consortium members and stakeholders without defining a formal advisory committee. To obtain input on the second topic, professors in the field of older adult care medicine, associated with older adults’ care networks, were invited to a scientific expert committee to guarantee adequate embedding of the Registry in the scientific world (Figure 1).

## Discussion

### Principal Findings

This paper describes how a data governance framework for the Registry Learning from Data in Nursing Homes has been developed. We used 8 governance principles, as described in the literature [26], to guide the developmental process. However, the governance principles were not fully self-explanatory, as the perspectives we gathered on the principles needed further elaboration before they could be readily used. Therefore, the development of the governance framework was furthermore based on stakeholder perspectives, legal advice, and consensus discussions between consortium partners.

We describe how the governing bodies, rules, and regulations of the framework are set up to reuse health data in an ethical, legal, and secure manner for research and quality improvement of care. The main finding is that stakeholders value the involvement of nursing home residents and their representatives, nursing home physicians, the boards of directors of nursing homes, and scientists and see this as a prerequisite for a trustworthy governance framework. Their involvement can be achieved by (1) a procedure in which residents can provide their consent or object for the reuse of the data, (2) transparency about the decisions that are made, and (3) giving stakeholders a position in a governing body. The governing bodies are responsible for developing and applying the criteria that third parties must satisfy to access all or a subset of the data set. Stakeholders stressed that assessment criteria should focus on the purpose of data reuse, methodological aspects, and type of organization requesting access to data from the Registry.

### Complexities in Exerting Control Over Medical Data

Legislation on the reuse of sensitive data, such as medical data, includes rules on whether a procedure is required for patients to exert control over the data. Patient control over health data reuse is not only needed to fulfill legal requirements but is also regarded as a facilitator to maximize the benefits for society. For example, Aitken et al (2019) describe a number of key principles in patient involvement and engagement, such as being transparent about the purpose of data reuse, being inclusive by taking patients’ views into account, and being accessible to a broad public by providing information in an understandable format [30]. In line with Aitken et al [30], stakeholders interviewed for the Registry Learning from Data in Nursing Homes mentioned as one of the main governance objectives, a 2-fold procedure in which nursing home residents and their representatives: (1) are informed about the aim and scope of the data reuse and (2) can make a well-informed decision on inclusion of the data in the Registry. However, stakeholders emphasize the complexity of providing understandable information to nursing home residents.

Literature on patients’ attitudes toward sharing health data for research purposes supports the view that patients’ willingness to share health data is affected by the information on data reuse [31]. Studies on informed consent by older people in general [32], older patients with dementia [33], and nursing home residents [34] mention that the process of informing older people and asking for their consent requires additional effort to provide understandable information so that they can make a well-informed decision on whether to share the data. In relation to this, stakeholders expressed concerns about the burden that informing residents and asking for their consent might place on both nursing home residents and health care professionals. This might differ between nursing homes depending on specific organizational characteristics, such as the nursing home population (the number of residents and their health status) and whether the procedure can be easily incorporated into an existing process (such as intake).

Therefore, the Registry was advised by the consulted lawyers to provide information on the applicable legal aspects to the boards of directors of nursing home organizations and then ask them if they support the implementation of a procedure in which residents and their representatives are asked for consent (opt-in procedure), or if they support a procedure in which all residents are included unless they have objected (opt-out procedure) based on specific organizational characteristics. For both procedures, information should be provided to every nursing home resident and representative, such as during the intake procedure. This requirement is part of the contract between a nursing home and the data processor (ie, Nivel). The Registry provides information materials, such as flyers that nursing home organizations have to spread among all residents and information that can be placed on the nursing home’s website. There is a risk that asking nursing home residents for consent might result in low consent rates and that this might increase selection bias compared with a procedure in which nursing home residents are included unless they have actively objected [35,36]. Therefore, for both procedures, we will monitor consent rates and, if possible, the characteristics of the nursing home residents included in the data.

### Ensuring Data Reuse Is in the Best Interests of Residents, Nursing Home Physicians, and Nursing Home Organizations

Stakeholders of the Registry Learning from Data in Nursing Homes emphasized the need to make health data available for secondary purposes that are in the best interests of residents and nursing home physicians in the nursing home sector and nursing home organizations, such as scientific research and providing feedback information to physicians. However, EHR data may also be useful for a broader range of secondary purposes. Incorporating the public’s and other stakeholders’ views on which purposes are in the best interest of the sector may stimulate the willingness to share data and may create support for the reuse based on the concept of “social license to operate.” Social license refers to informal acceptance and permission for researchers or organizations to access and reuse health data for certain purposes, as its benefits are generally acknowledged [37].

In this study, concerns were raised that stakeholders’ support for the Registry would decline if data were provided to organizations with an inspectorate task, organizations to which the Wob applies, and organizations with a commercial interest. Studies on patients’ attitudes toward data sharing for research found that patients are more willing to share EHR data if there is a guarantee that the data will be used for research in the public interest [10]. The findings of a study on public attitudes toward commercial access to health data showed that this guarantee, in combination with security and privacy measures, also supports patients’ willingness to share data with most commercial organizations within the health sector [38]. Being transparent about the purposes of data reuse, the parties that are allowed access to the data, and the data processing methods might help generate trust in the integrity of a registry [10,38].

Several measures will enhance the integrity of the Registry. First, requests for data will be reviewed by a committee that includes a representation of nursing home physicians, nursing home residents, and boards of directors of nursing homes. Second, the committee will use a set of assessment criteria that include the purpose of data use and the characteristics of the requesting party. Third, the information included in the Registry will be limited to data that nursing home physicians consider necessary for providing basic care. Data access for third parties will be limited to a subset of the data set, as needed for the research. Fourth, the assessment procedure and granted requests will be published on the website. These measures are included in the Registry’s governance framework with the aim of earning and maintaining the trust of nursing home residents, other stakeholders, and the public that the data will be used in the best interests of residents, nursing home physicians, and boards of directors of nursing homes.

### Taking the Organizational Context Into Account

The Registry’s data governance framework was established for an interorganizational environment. First, Registry Learning from Data in Nursing Homes was initiated by a consortium of 3 organizations (Textbox 1), which share accountability and responsibilities. Second, the context included almost 1800 nursing home physicians working for more than 300 different nursing home organizations in the Netherlands and recording health data in different EHR systems [19]. Although the principles and governance framework of Willison et al [26] are also set up for an interorganizational environment, their framework does not consider the environment and its consequences. In the case of the Registry, the 3 consortium partners signed a contract documenting the accountability and responsibilities of each partner. Furthermore, as there are several different EHR systems in use in Dutch nursing homes, data recording processes and other procedures may differ between nursing homes. Therefore, the governance framework of the Registry includes decisions on data exchange standards. A data exchange standard improves the usability of the data for knowledge development, ie, “fitness for purpose” or “data quality.” Fitness for purpose is one of the challenges in a learning health system [4,39]. Especially in the Dutch nursing home setting, where standardization of EHRs is at an early stage, standardization is an aspect that requires particular attention. As a data governance framework provides the opportunity to make decisions on roles and responsibilities for standardization and to establish procedures to monitor fitness for a purpose, standardization may be considered as part of a governance framework. For this reason, the requirements for data exchange [40,41] and the organizational context [41] should be considered in the governance framework.

### Methodological Considerations

The methodology used in this study had several strengths. First, this is one of the few studies to explore and describe the expectations and needs of stakeholders with regard to data governance in the nursing home sector. The development of the governance framework for Registry Learning from Data in Nursing Homes was an extensive and time-consuming process. The development process required several discussions to reach a consensus on the governing bodies, rules, and regulations of the framework. However, using different sources (ie, interviews, legal advice, expertise regarding other governance frameworks, and consensus discussions) helps gather the information needed to balance the different possibilities for the framework. This study serves as a real-world example of how a governance framework can be developed within the health care sector and therefore contributes to the knowledge gap mentioned in the introduction. Second, we interviewed a broad range of stakeholders to gain insights into the different perspectives and expectations regarding the governance framework of the Registry. Although we interviewed a broad range of stakeholders, it was not possible to interview all stakeholders. Therefore, 20 individuals were selected for this study. Another limitation that should be kept in mind is that the governance framework of the Registry Learning from Data in Nursing Homes is based on the Dutch legal framework. The GDPR is applicable to all European Union member states. However, as the implementation of the GDPR differs between member states [24], initiatives that want to set up a data governance framework in another country should carefully explore the implications of legislation.

### Future Implications

On the basis of the results of this study, we recommend that future research and policies focus on the following 3 topics. First, an important goal of data governance is to build public trust and increase the trust of health care providers in the appropriate use of health data for secondary purposes. Research on data governance and public attitudes toward reusing health data have provided inputs that could help reach this goal. We recommend future research on whether the implemented data governance framework furthers this goal. Second, there is considerable debate at present about which consent procedure (opt in or opt out) is required by law in what situation and what effect the consent procedure will have on selection bias. It is important that future research provides a better understanding of the consequences of each procedure on the usability of health data for secondary purposes in the nursing home sector. Third, any governance framework is developed based on current circumstances, such as the available technologies and legislation on the use of EHR data for secondary purposes. As there is a growing interest in the effects of legislation on reusing health data, legislation might change in the future. In addition, technologies increasingly allow decentralized databases to replace centralized databases. A governance framework should respond efficiently and effectively to changes in these circumstances. Future research could identify the consequences of changes in data governance rules and regulations and how a governance framework can respond to these changes.

### Conclusions

We developed a data governance framework for Registry Learning from Data in Nursing Homes, which collects data recorded by nursing home physicians in the EHRs of nursing homes in the Netherlands. The development of the governance framework was an iterative process guided by 8 governance principles and the outcome of several consensus discussions between consortium members. The results from interviews with stakeholders, legal advice, and consortium members’ knowledge and expertise regarding other governance frameworks were taken together. This input served to inform the application of governance principles in the particular contexts of both the nursing home sector and the Netherlands. Many different stakeholders were involved in the development process and will continue to be involved in governing bodies responsible for implementing the rules and regulations of the governance framework. Engagement of the full range of stakeholders in an early stage of the governance framework development process helps align the framework with the perspectives of stakeholders, build trust, and generate support for the Registry. Although this study focuses on the reuse of data from EHRs pertaining to nursing home residents, we believe that the findings are also useful for the development of governance frameworks for the reuse of routine health data in other settings.

## Appendix

Multimedia Appendix 1 Presentation used during the stakeholder interviews (translated from Dutch to English).

## Abbreviations

EHR: electronic health record
GDPR: General Data Protection Regulation
IGJ: Dutch Health and Youth Care Inspectorate
UAVG: Dutch GDPR Implementation Act
WGBO: Dutch Medical Treatment Contracts Act
Wob: Dutch Act on Public Access to Government Information

## Data Availability

Anonymized data generated and analyzed during this study are not publicly available owing to reasons of confidentiality but are available from the corresponding author upon reasonable request.
