# Supplementary material for: Setting up a Governance Framework for Secondary Use of Routine Health Data in Nursing Homes: Development Study Using Qualitative Interviews
Source: J Med Internet Res. 2023 Jan 25;25:e38929. doi: 10.2196/38929 (PMC9909520; doi:10.2196/38929)
Supplement: Multimedia Appendix 1 [file jmir_v25i1e38929_app1.pptx]

## Slide 1
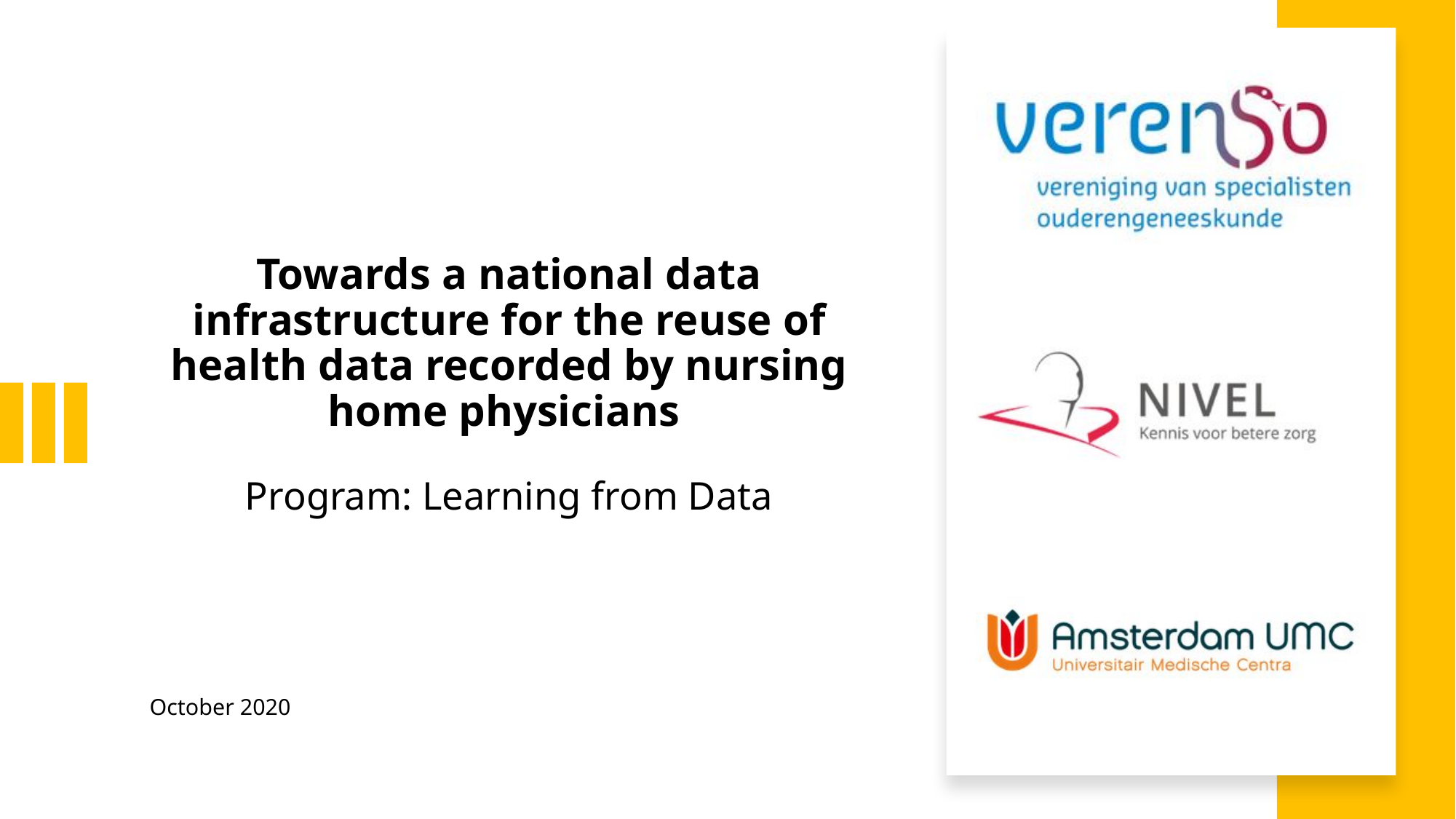

# Towards a national data infrastructure for the reuse of health data recorded by nursing home physicians Program: Learning from Data
 October 2020

## Slide 2
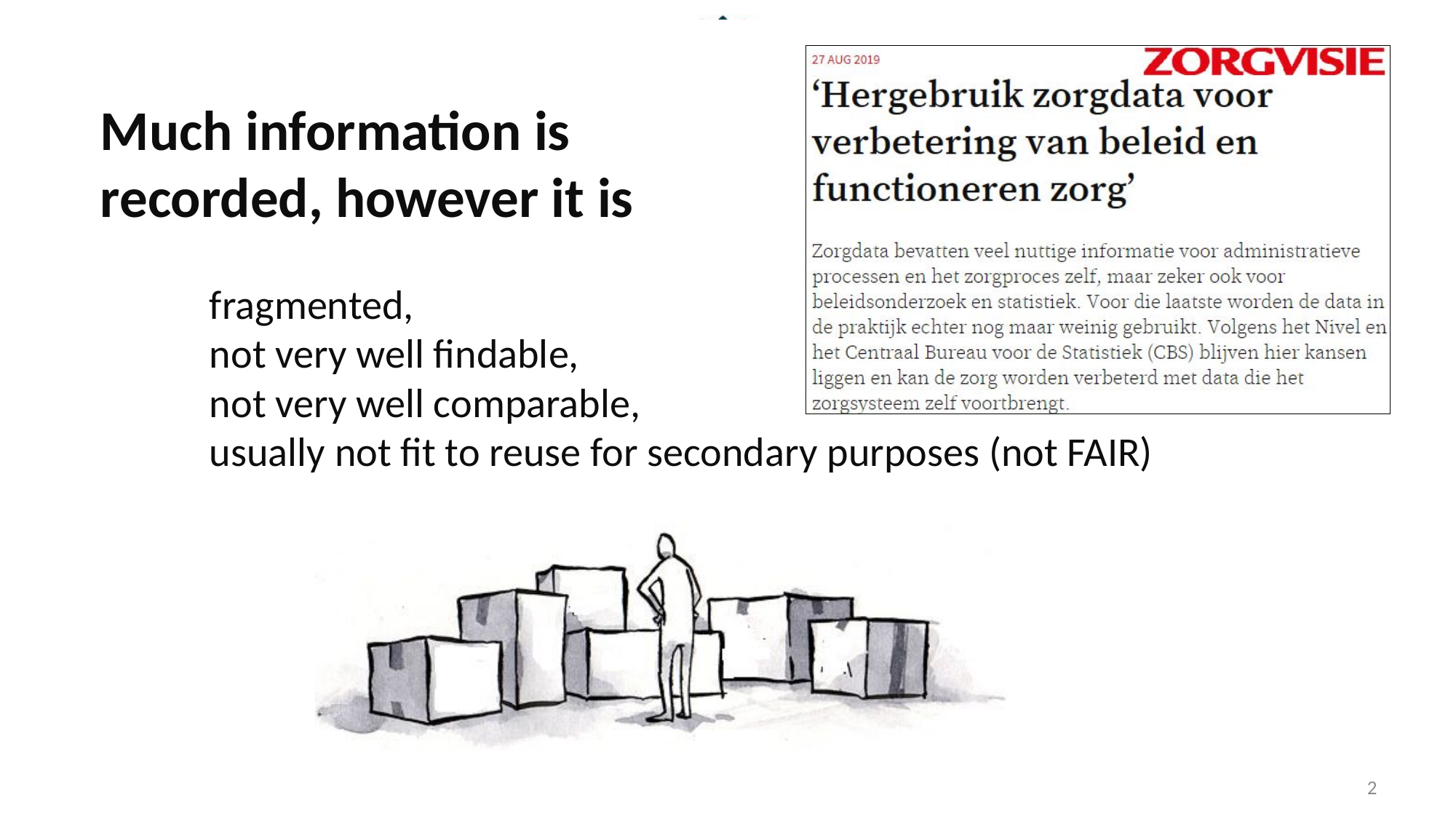

Much information is
recorded, however it is
	fragmented,
	not very well findable,
	not very well comparable,
	usually not fit to reuse for secondary purposes (not FAIR)
2

## Slide 3
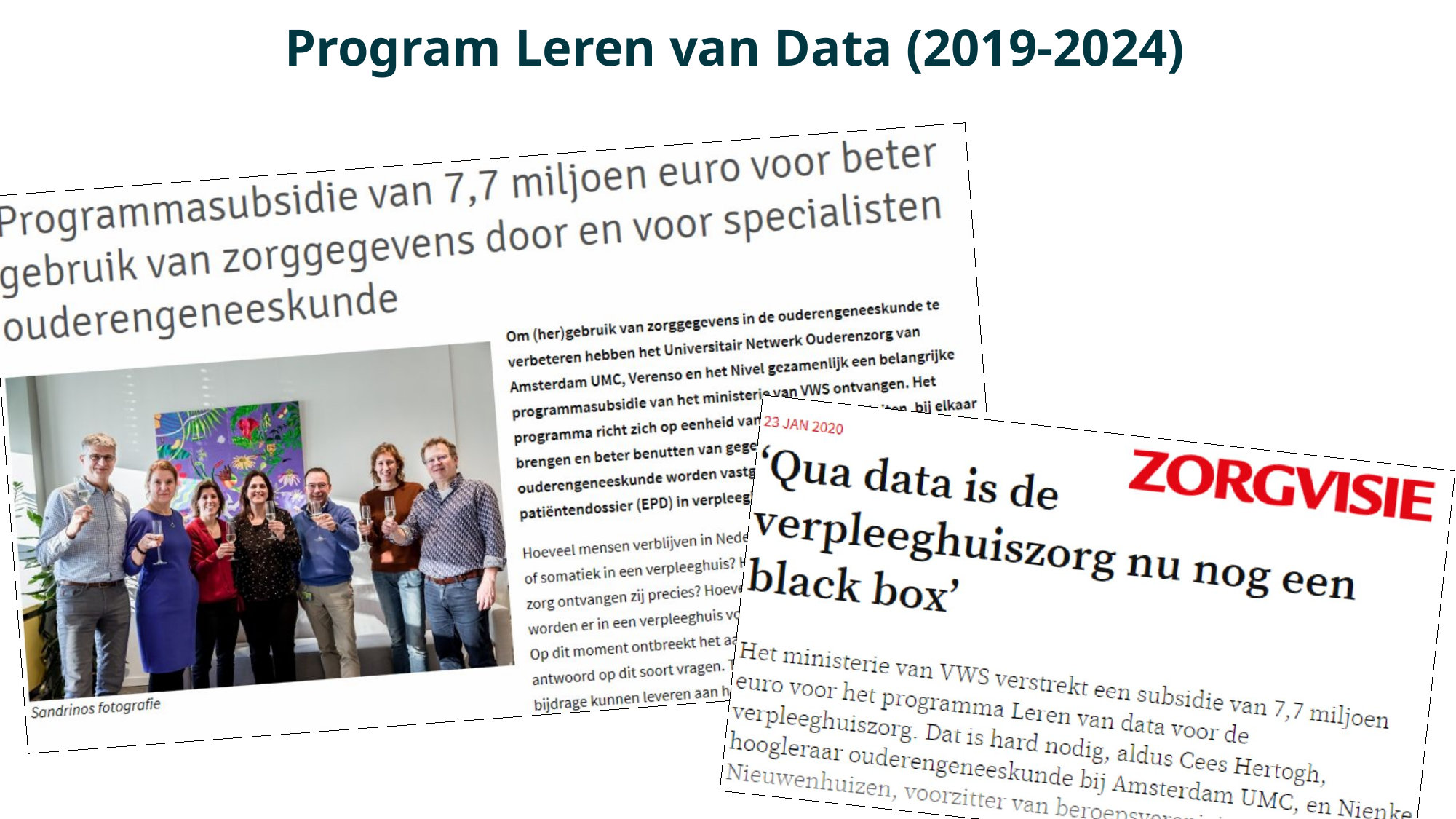

Program Leren van Data (2019-2024)
3

## Slide 4
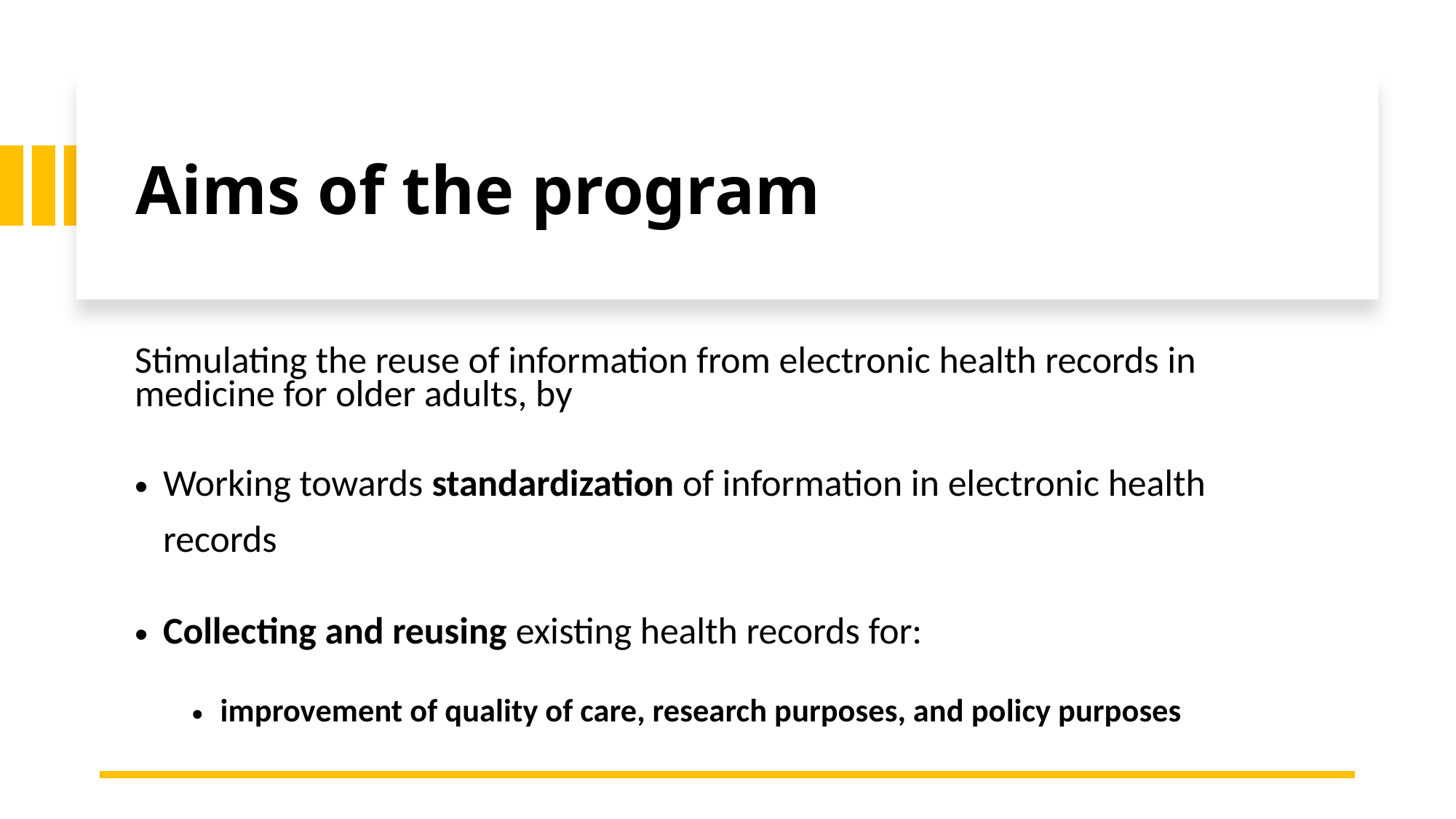

# Aims of the program
Stimulating the reuse of information from electronic health records in medicine for older adults, by
Working towards standardization of information in electronic health records
Collecting and reusing existing health records for:
improvement of quality of care, research purposes, and policy purposes

## Slide 5
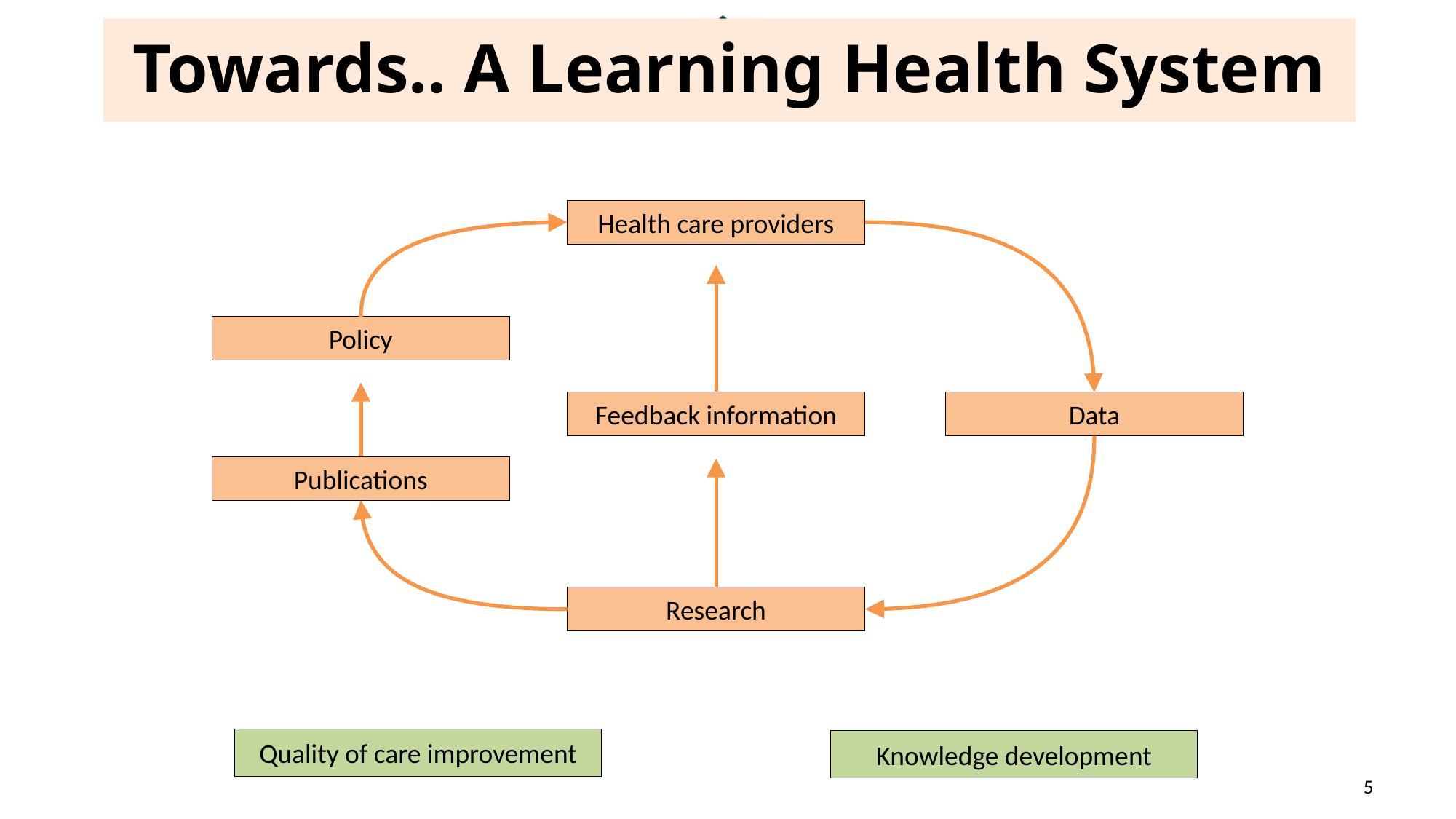

Towards.. A Learning Health System
Health care providers
Policy
Feedback information
Data
Publications
Research
Quality of care improvement
Knowledge development
5

## Slide 6
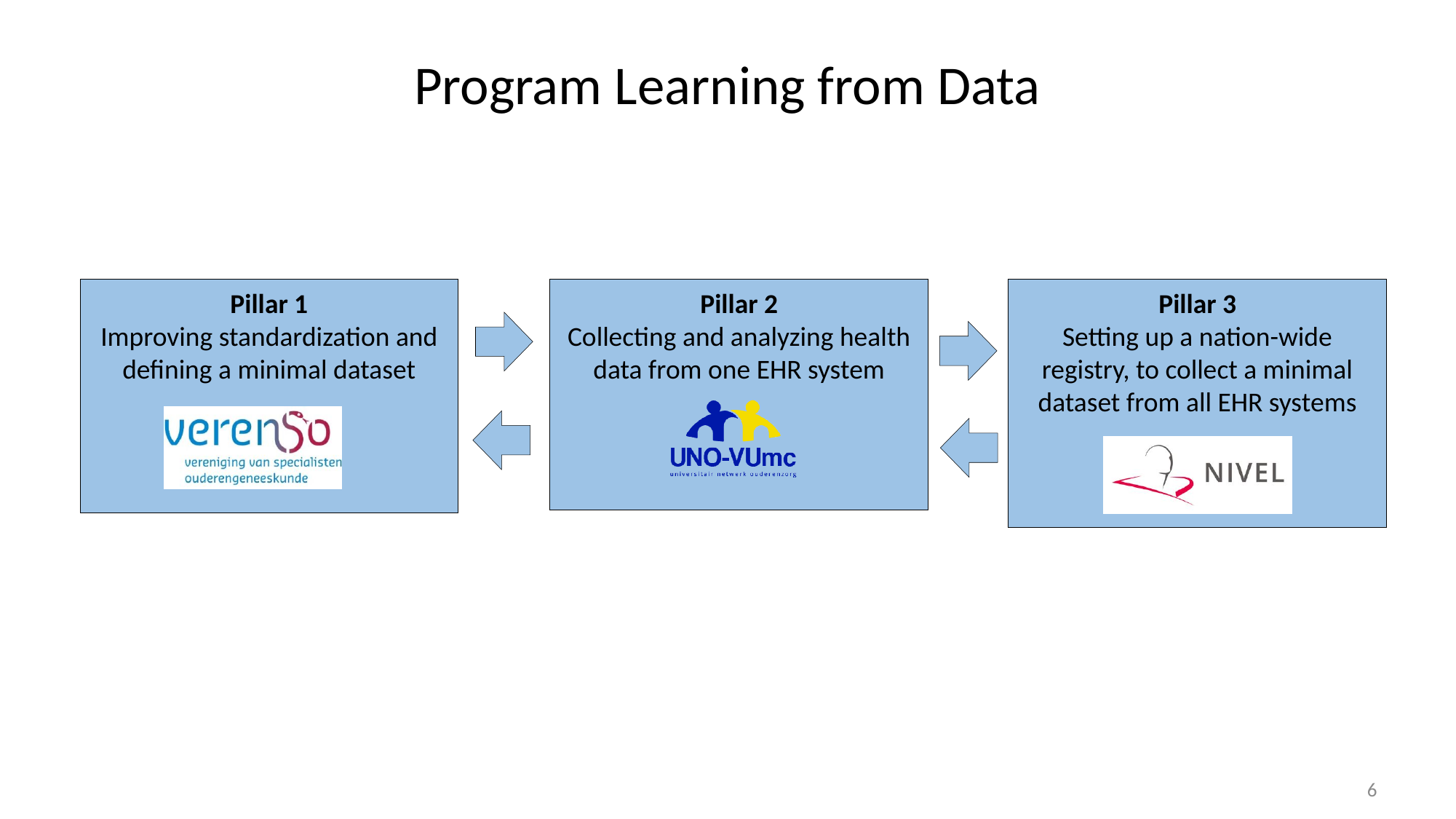

Program Learning from Data
Pillar 1
Improving standardization and defining a minimal dataset
Pillar 2
Collecting and analyzing health data from one EHR system
Pillar 3
Setting up a nation-wide registry, to collect a minimal dataset from all EHR systems
6

## Slide 7
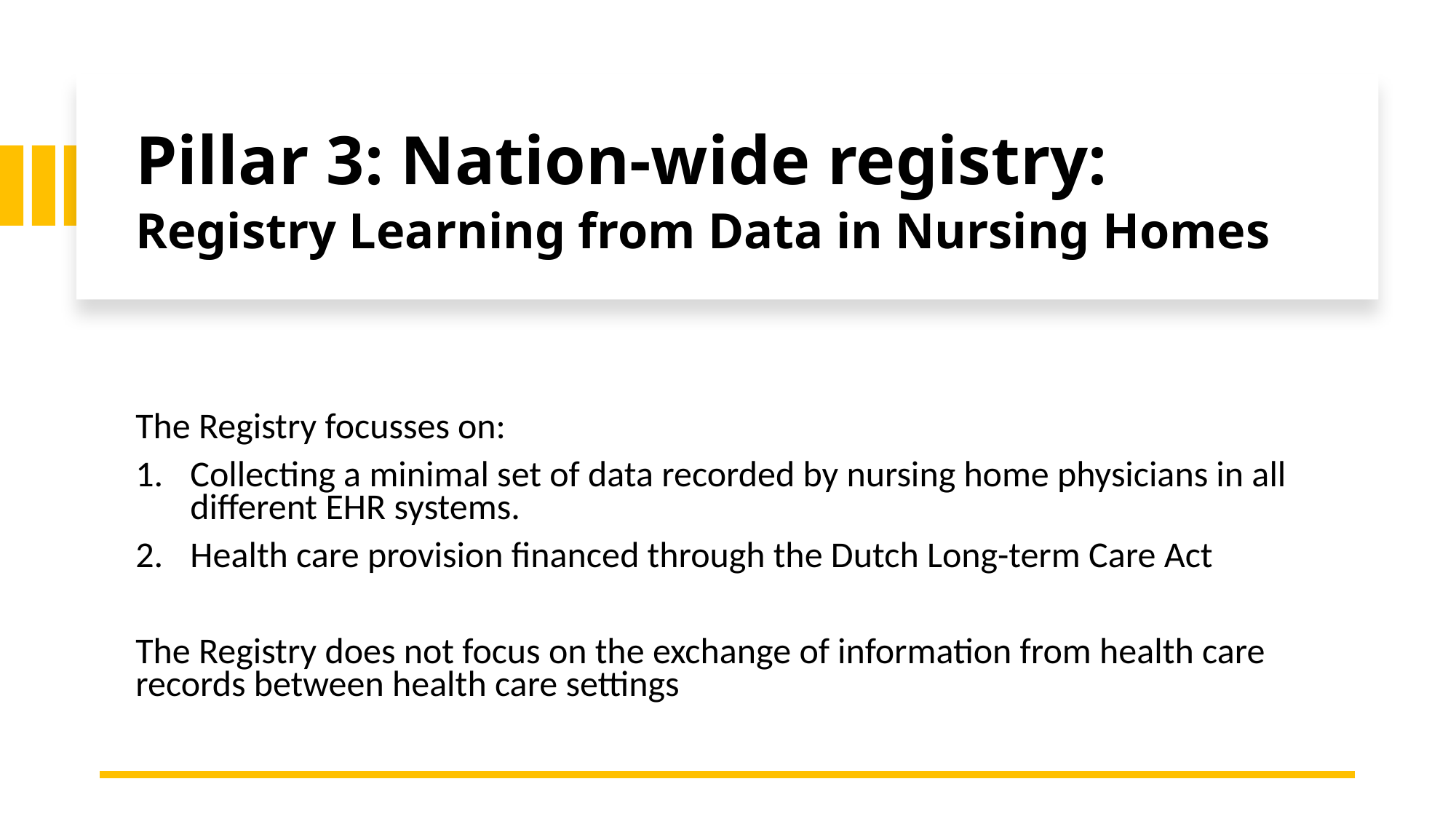

# Pillar 3: Nation-wide registry: Registry Learning from Data in Nursing Homes
The Registry focusses on:
Collecting a minimal set of data recorded by nursing home physicians in all different EHR systems.
Health care provision financed through the Dutch Long-term Care Act
The Registry does not focus on the exchange of information from health care records between health care settings

## Slide 8
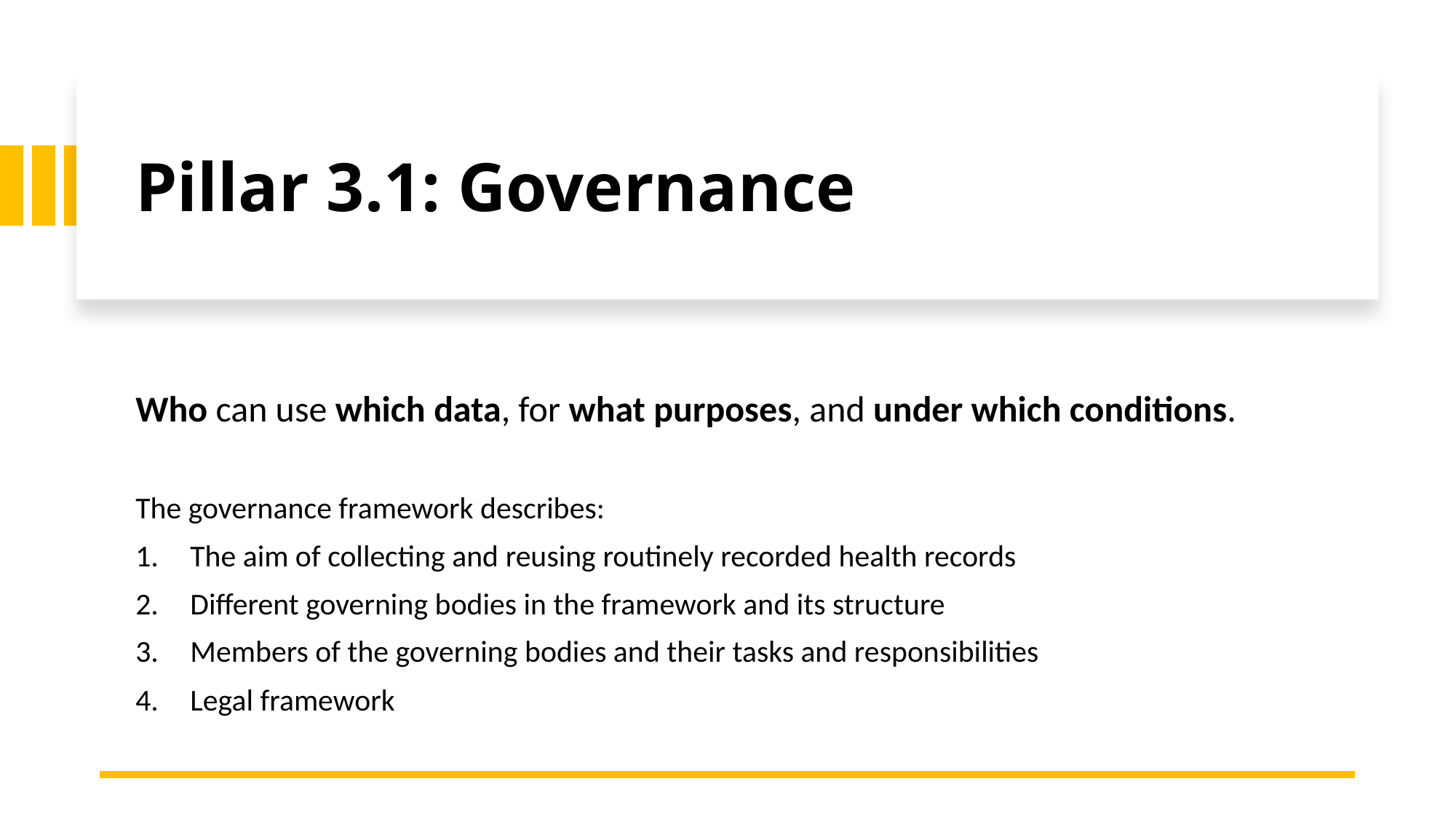

# Pillar 3.1: Governance
Who can use which data, for what purposes, and under which conditions.
The governance framework describes:
The aim of collecting and reusing routinely recorded health records
Different governing bodies in the framework and its structure
Members of the governing bodies and their tasks and responsibilities
Legal framework

## Slide 9
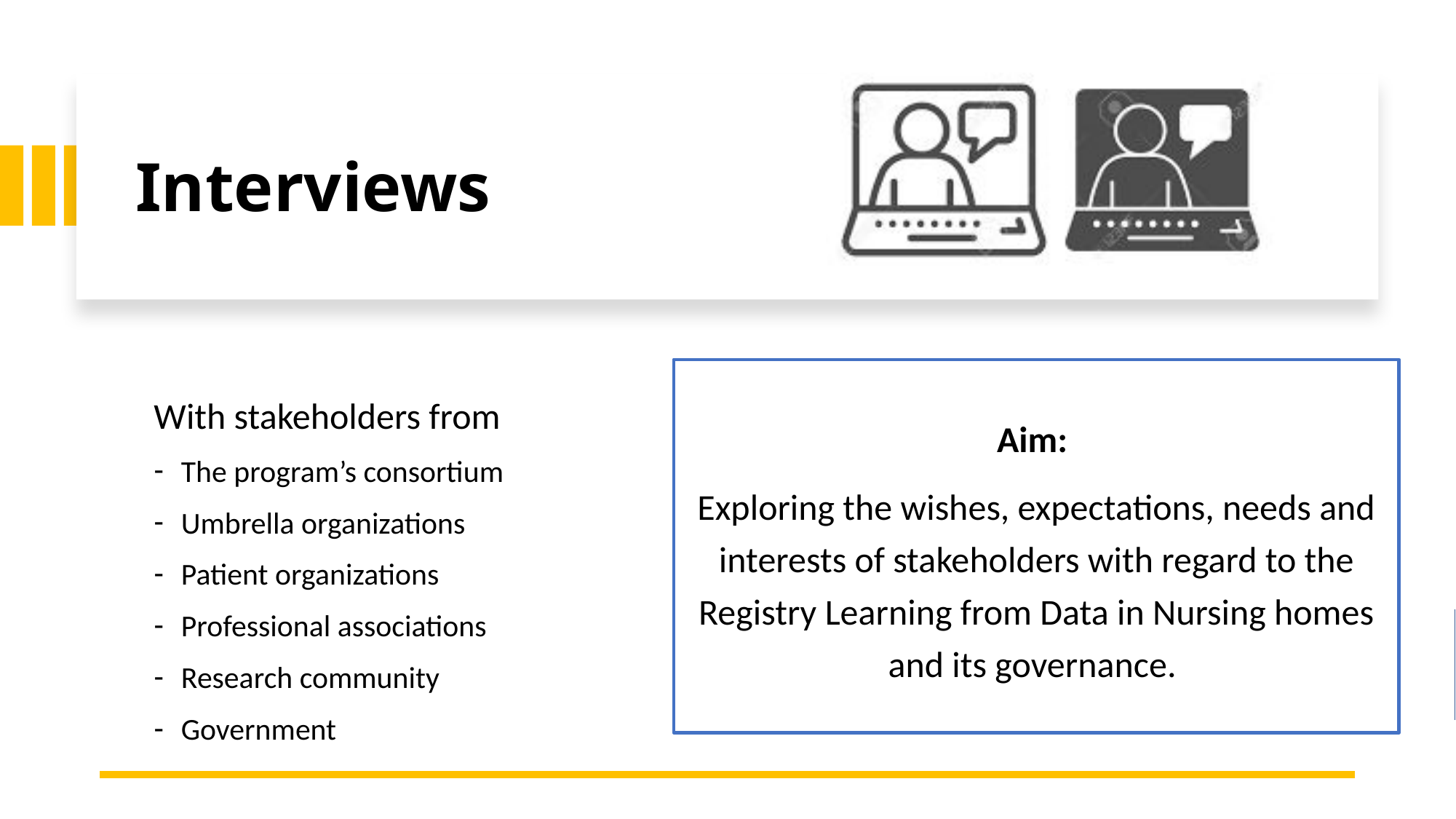

# Interviews
Aim:
Exploring the wishes, expectations, needs and interests of stakeholders with regard to the Registry Learning from Data in Nursing homes and its governance.
With stakeholders from
The program’s consortium
Umbrella organizations
Patient organizations
Professional associations
Research community
Government

## Slide 10
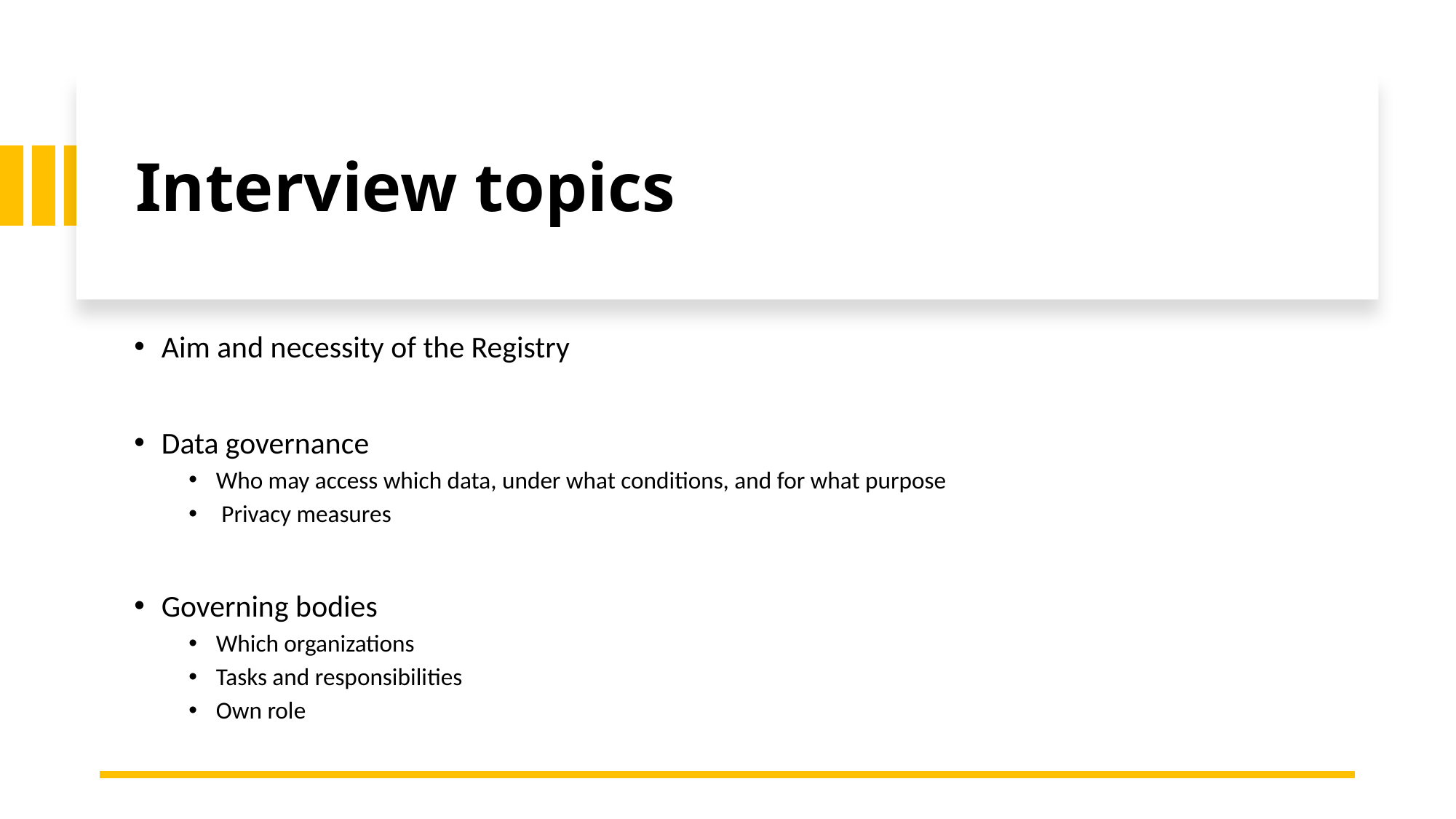

# Interview topics
Aim and necessity of the Registry
Data governance
Who may access which data, under what conditions, and for what purpose
 Privacy measures
Governing bodies
Which organizations
Tasks and responsibilities
Own role
